# Expert Opinion on the Use of Novel Oral Anticoagulants for Stroke Prevention in Non-valvular Atrial Fibrillation for the Primary Care Setting in India: A Literature Review

**DOI:** 10.7759/cureus.25102

**Published:** 2022-05-18

**Authors:** Jamshed Dalal, Fali Poncha, Sandeep Bansal, Arvind Das, Praveen Gupta, Debasis Ghosh, Anshu Rohatgi, Murugesh S Hiremath, Kartikeya Bhargava, Arun Gopi, Mithun Mali

**Affiliations:** 1 Cardiology, Kokilaben Hospital, Mumbai, IND; 2 Neurology, Jaslok Hospital, Mumbai, IND; 3 Cardiology, Vardhman Mahavir Medical College and Safdarjung Hospital, Delhi, IND; 4 Cardiology, Max Hospital, Gurgaon, IND; 5 Neurology, Fortis Hospital, Gurgaon, IND; 6 Cardiology, Apollo Gleneagles Hospital, Kolkata, IND; 7 Neurology, Sir Ganga Ram Hospital, New Delhi, IND; 8 Cardiology, Ruby Hall Clinic, Pune, IND; 9 Cardiology, Medanta - The Medicity, Gurgaon, IND; 10 Cardiology, Metromed International Cardiac Centre, Kozhikode, IND; 11 Internal Medicine, Vijayalakshmi Hospital, Bengaluru, IND

**Keywords:** frail elderly, renal insufficiency, hemorrhage, stroke, atrial fibrillation

## Abstract

Atrial fibrillation (AF), the most prevalent cardiac arrhythmia encountered in clinical practice, is linked with substantial morbidity and mortality due to accompanying risk of stroke and thromboembolism. Patients with AF are at a five-fold higher risk of suffering from a stroke. Anticoagulation therapy, with either vitamin K antagonists or novel oral anticoagulants (NOACs), is a standard approach to reduce the risk. Consultant physicians (CPs) in India are the primary point of contact for the majority of patients before they approach a specialist. The CPs may face challenges in screening and diagnosing AF patients. The apprehensions associated with managing AF patients with anticoagulants, further add to the challenges of a CP. This review aimed to identify the key decision points for the CPs to diagnose AF and initiate anticoagulation in patients with non-valvular AF (NVAF) and bring to the table a simplified recommendation supported by expert opinion and guidelines for stroke prevention in NVAF patients.

## Introduction and background

Atrial fibrillation (AF) is the most prevalent cardiac arrhythmia encountered in clinical practice [1]. The estimated global prevalence of AF in 2010 was reported to be nearly 33.5 million individuals [2]. The World Heart Federation (WHF) in its 2020 update of the "Roadmap" initiative reported the prevalence of AF in India to be between 0.1% and 0.5% in the general population for the period of 2001-2010. The prevalence for the general population between 2011 and 2020 was estimated to be 1.6% and 5.6%, respectively, in the population aged ≥75 years [3]. The Indian Heart Rhythm Society (IHRS) AF registry revealed that Indian patients with AF are more than a decade younger than those in the Western world. The underlying cause in Indian patients was determined to be rheumatic valvular heart disease (RHD), followed by hypertension, diabetes, and coronary artery disease [4].

AF is associated with substantial mortality and morbidity from stroke and thromboembolism [5]. AF patients pose a five-fold higher risk of stroke [6]. The mortality associated with ischemic stroke can be nearly twice in patients with AF as compared to those without AF [7]. Traditionally, vitamin K antagonists (VKA), especially warfarin were the preferred anticoagulants. But due to their many limitations including narrow therapeutic window, need for regular monitoring, slow onset of action, and numerous drug and food interactions, novel oral anticoagulants (NOACs) have evolved as the preferred agents [8]. The time in therapeutic range (TTR) is a quality measure commonly used for the assessment of anticoagulation therapy with warfarin and it co-relates with improved patient outcomes for patients with AF treated with warfarin [9]. Apixaban for Reduction in Stroke and Other Thromboembolic Events in Atrial Fibrillation (ARISTOTLE) study and Randomized Evaluation of Long-Term Anticoagulation Therapy (RE-LY) study, which compared apixaban and dabigatran, respectively, with warfarin for stroke prevention in AF (SPAF), had the lowest TTR recorded from India [10].

Consultant physicians (CPs) are the primary point of contact for patients. In the opinion of the experts, the major issues faced in India are the lack of adequate training for screening and diagnosis of AF. Additionally, due to apprehensions related to the risk of bleeding, the CPs are hesitant to initiate oral anticoagulation. Available evidence suggests that patients with AF, stroke, or transient ischemic attack were found to be undertreated with oral anticoagulation therapy in the majority of studies [5]. The authors in this review aim to identify the main points for the CPs to identify AF and initiate anticoagulation in patients with non-valvular AF (NVAF) and bring to the table a simplified recommendation supported by expert opinion and guidelines for stroke prevention in NVAF patients.

All the experts who were invited for the preceding advisory board are of ~20 to 30 years of clinical experience in the management of NVAF and are very well aware of the evolving landscape of anticoagulants. Their expert opinion was weighed along with the evidence from the literature including the recommendations from the guidelines to propose the protocol for the management of stroke prevention in AF suitable for the primary care setting in India.

## Review

Recent guidelines

Various clinical practice guidelines have provided recommendations for the prevention of stroke in patients with AF. The recommendations made by the 2019 American College of Cardiology/American Heart Association Task Force on Clinical Practice Guidelines and the Heart Rhythm Society (AHA/ACC/HRS Focused Update of the latest AHA/ACC/HRS Guideline) [11], the 2020 European Society of Cardiology (ESC) [12], and the 2021 European Heart Rhythm Association Practical Guide [13] are summarized in Table 1.

**Table 1:** Summary of guidelines AF: atrial fibrillation; CHA_2_DS_2_-VASc: congestive heart failure, hypertension, age ≥75 (doubled), diabetes, stroke (doubled), vascular disease, age 65-74, and sex (female); INR: international normalized ratio; NOAC: novel oral anticoagulants; OAC: oral anticoagulation; VKA: vitamin K antagonists The table is adapted from the 2019 American College of Cardiology/American Heart Association Task Force on Clinical Practice Guidelines and the Heart Rhythm Society (AHA/ACC/HRS Focused Update of the latest AHA/ACC/HRS Guideline) [11], the 2020 European Society of Cardiology (ESC) [12], and the 2021 European Heart Rhythm Association Practical Guide [13].

Guideline recommendations	
Anticoagulants are recommended	In AF patients, oral anticoagulants are recommended when a CHA_2_DS_2_-VASc score of ≥2 in men or ≥3 in women. (Level of evidence: I; strength of recommendation: A)
	NOACs are preferred over warfarin in NOAC-eligible patients with AF (exception: patients with “moderate-to-severe mitral stenosis or a mechanical heart valve”)
Pointers to be noted before initiating anticoagulants	Choice of OACs should be based on thromboembolism risk, irrespective of the AF pattern
	Evaluate renal function and hepatic function before starting NOAC therapy and should be reevaluated at least once a year
	Reevaluate the need for and choice of anticoagulant therapy periodically
	Reassess stroke and bleeding risks
	Use of antiplatelet monotherapy or aspirin-clopidogrel combination is discouraged
Contraindications of NOACs	Patients with mechanical prosthetic valve and moderate to severe mitral stenosis
	Patients with bioprosthetic valve can be anticoagulated with NOACs only if it is degenerative mitral regurgitation or if the valve is in the aortic position
	Patients with hypertrophic cardiomyopathy can be considered for NOAC treatment but the data is limited

Protocol for consulting physicians

Screening

In 20-45% of stroke cases, the underlying AF is detected at the time of stroke. The reason for under-diagnosis of AF can be because of a significant proportion of cryptogenic strokes attributed to undetected AF. AF remains asymptomatic in almost one-third of the cases and most of the symptomatic patients have atypical symptoms [14]. Paroxysmal AF can progress to sustained forms of AF within the first year and the progression rate ranges from 8.6% to 15% [15]. Unattended sustained AF has a high risk of stroke and heart failure (HF). Hence, for many patients, the steps required to prevent sustained AF should begin early. Stroke is preventable with a 66% reduction in risk when appropriate anticoagulation therapy is given to eligible patients with AF. Hence screening of patients for AF is vital to prevent stroke in patients [14].

**Figure 1:**
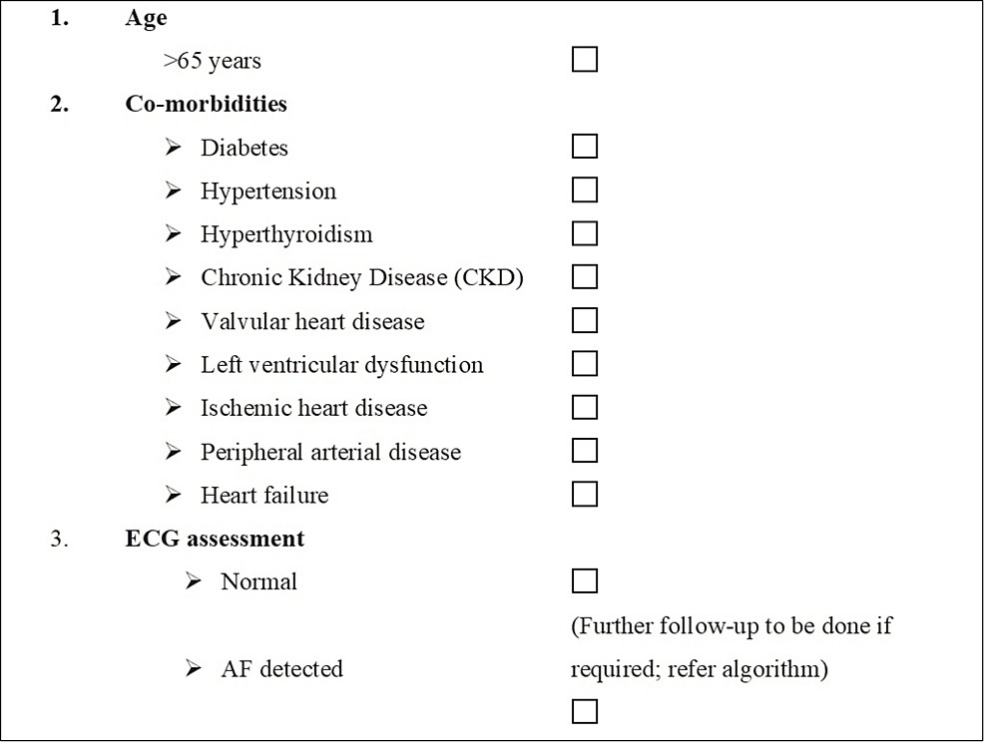
Panel recommendations for screening of high-risk patients for AF AF: atrial fibrillation; CKD: chronic kidney disease; ECG: electrocardiogram

The conventional risk factors for AF include coronary heart disease, hypertension, heart failure, left ventricular diastolic dysfunction, diabetes, hyperthyroidism, obesity, and valvular heart disease [16]. The experts in the advisory board opined that CPs should assess the high-risk patients for AF and can follow a checklist to identify patients (Figure 1). The panelists suggested that if the electrocardiogram (ECG) is normal, the patients should be followed up for their primary condition for which they visited. The CPs can consider doing echocardiography if the patient has cardiac co-morbidities.

Diagnosis

AF is suspected when an irregular pulse is observed in a patient and must be confirmed using a 12-lead ECG [17]. The diagnosis of AF requires documentation of ECG recorded cardiac rhythm showing the typical pattern of AF where one episode lasts for at least 30 seconds. When clinical suspicion of atrial fibrillation persists despite normal ECG, a Holter monitor (24-hour recording) or event monitor (seven to 30-day recording) may be warranted [17]. There are five types of AF identified based on the presentation, duration, and spontaneous termination of AF episodes (Table 2) [12]. The anticoagulation strategy however does not depend upon the type of AF.

**Table 2:** Types of atrial fibrillation AF: atrial fibrillation The table is adapted from Hindricks et al., (2021; ESC 2020) [12].

Sr. no.	Type of AF	Presentation
1.	First diagnosed AF	Previous diagnosis not established regardless of AF duration or severity
2.	Paroxysmal AF	AF episodes that terminate within 7 days (spontaneously or with cardioversion)
3.	Persistent AF	AF episode lasting for more than 7 days. This definition is inclusive of episodes terminated by cardioversion (with drugs or by direct current cardioversion) after 7 days or more
4.	Long-standing persistent AF	AF which continues for >12 months before rhythm control is implemented
5.	Permanent AF	AF accepted by both: the patient and the physician; rhythm control not implemented.

*Management* 

The congestive heart failure, hypertension, age ≥75 (doubled), diabetes, stroke (doubled), vascular disease, age 65-74, and sex (female) (CHA_2_DS_2_-VASc) score is used to identify the risk of thromboembolism in AF patients and hypertension, abnormal renal and liver function, stroke, bleeding, labile INR, elderly, drugs or alcohol (HAS-BLED) score assesses the risk of bleeding in anticoagulation (Table 3) [18,19].

**Table 3:** Calculation of CHA2DS2-VASc score and HAS-BLED score *HAS-BLED score of ≥3 warrants regular clinical review and follow-up. CHA_2_DS_2_-VASc: congestive heart failure, hypertension, age ≥75 (doubled), diabetes, stroke (doubled), vascular disease, age 65-74, and sex (female); HAS-BLED: hypertension, abnormal renal and liver function, stroke, bleeding, labile INR, elderly, drugs or alcohol; PAD: peripheral arterial disease; MI: myocardial infarction; TIA: transient ischemic attack; TE: transesophageal echocardiography; LV: left ventricular; BP: blood pressure; INR: international normalized ratio: NSAIDs: non-steroidal antiinflammatory drugs The table is adapted from CHA_2_DS_2_-VASc 1 [18] and HAS-BLED Tool (2012) [19].

Stroke risk factors	Score
CHA_2_DS_2_-VASc score	
Congestive heart failure/LV dysfunction	1
Hypertension	1
Aged ≥ 75 years	2
Diabetes mellitus	1
Stroke/ TIA/ TE	2
Vascular disease (prior MI, PAD, or aortic plaque)	1
Aged 65-75 years	1
Sex category (i.e., female gender)	1
Maximum score	9
HAS-BLED score *	
Hypertension, i.e., uncontrolled BP	1
Abnormal renal/liver function	1 or 2
Stroke	1
Bleeding tendency or predisposition	1
Labile INR	1
Age (e.g., > 65)	1
Drugs (e.g., concomitant aspirin or NSAIDs) or alcohol	1
Maximum score	9
HAS-BLED score of ≥3 warrants for regular clinical review and follow-up	

Evolution of treatment

**Figure 2:**
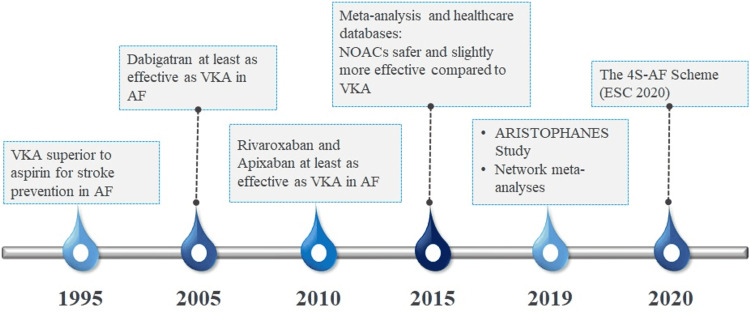
Evolution of treatment for stroke prevention in AF VAK: vitamin K antagonists; NOACs: novel oral anticoagulants; 4S-AF: stroke risk, symptoms, severity of AF burden, and substrate; AF: atrial fibrillation; ESC: European Society of Cardiology; ARISTOPHANES: Anticoagulants for Reduction in Stroke: Observational Pooled Analysis on Health Outcomes and Experience of Patients

The management strategies for stroke prevention have evolved substantially in the past three decades. Novel drugs have been developed and robust clinical studies and meta-analyses have supported the use of anticoagulants for stroke prevention. Some of the landmark events in the evolution of stroke prevention management have been presented in Figure 2 [12,20]. 

Issues with warfarin

Warfarin, a vitamin K antagonist is the most widely used anticoagulant. However, in the recent past, its use for the same has decreased due to many challenges including unreliable INR values, unpredictable outcomes, and need for continuous monitoring and availability of NOACs [21]. Warfarin loading doses may result in a hypercoagulable state and potential clot formation because of significant reductions in protein C and protein S levels (Table 4) [22].

**Table 4:** Advantages of NOACs over VKA in the management of stroke prevention in patients with AF The table mentions the limitations of VKA in stroke prevention in AF patients and its implications on clinical practice which are addressed by the NOACs [23,24]. *As per the expert opinion. AF: atrial fibrillation; NOAC: novel oral anticoagulant; VKA: vitamin K antagonists

Limitations of VKA	Implications on clinical practice*	Advantages of NOACs
Risk of bleeding complications, including intracranial hemorrhage	Increased hospital cost, increased hospitalization rate, increased mortality	Lower incidence of major bleeding
Routine monitoring required	Increased laboratory cost, increased hospital visits	Convenience of use, no need for laboratory monitoring
Dose adjustments frequently needed	Increased hospital visits	NOACs are administered in fixed doses, except when a patient has a disorder of the liver or kidney
Slow onset of action	Increased risk of stroke	Rapid onset and offset of action, a short half-life
Narrow therapeutic window	Increased risk of stroke and bleeding	Wide therapeutic window
Dietary restrictions Numerous drug interaction	Reduced adherence, increased risk of stroke and bleeding	Fewer drug and food interactions
Variability in patient response	Difficulty in standardized approach and frequent follow-ups	Less variability

**Figure 3:**
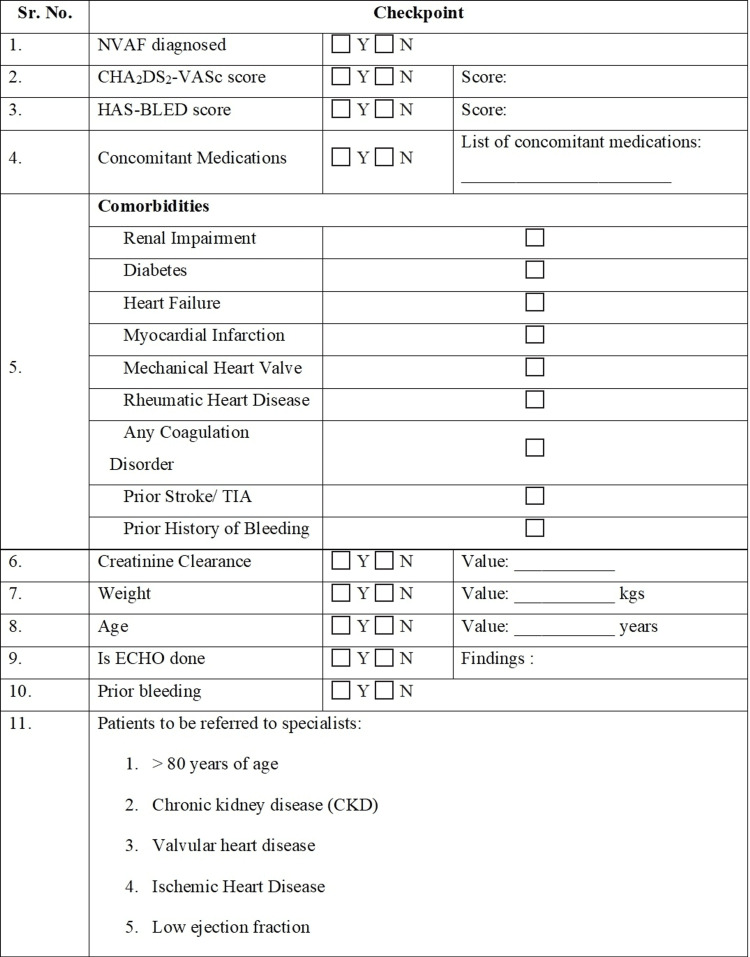
Checklist for CPs before initiating OAC therapy or referring to a specialist CKD: chronic kidney disease; ECHO: echocardiogram; MI: myocardial infarction; NVAF: non-valvular atrial fibrillation; OAC: oral anticoagulant; PCI: percutaneous coronary intervention; TIA: transient ischemic attack

In a meta-analysis by Ruff et al., the safety and efficacy of NOACs were compared to warfarin in patients with atrial fibrillation. NOACs were associated with a reduced composite of stroke or systemic embolic events by 19% as compared with warfarin. There was also a reduction in major bleeding by 14% with NOACs [25]. In the Anticoagulants for Reduction in Stroke: Observational Pooled Analysis on Health Outcomes and Experience of Patients (ARISTOPHANES) study, warfarin was reported to have a higher stroke/systemic embolism rate as compared to apixaban (1.92 vs. 1.33 per 100 person-years), dabigatran (1.74 vs. 1.44 per 100 person-years), and rivaroxaban (1.90 vs. 1.51 per 100 person-years). Apixaban (hazard ratio {HR}, 0.60; 95% confidence interval {CI}, 0.56-0.63) and dabigatran (HR, 0.71; 95% CI, 0.65-0.78) were associated with lower rates of major bleeding as compared to warfarin, while rivaroxaban (HR, 1.06; 95% CI, 1.02-1.10) had a higher rate of major bleeding compared with warfarin [26]. Apixaban was associated with fewer major bleeds in comparison to all other NOACs. Figure 3 depicts the checklist for CPs designed based on the expert opinion of the advisors.

Tailor-Made Therapy

Elderly and very elderly: Elderly patients (age above 75 years) [27] are more prone to thromboembolic events as well as have an increased risk of bleeding [28]. The factors apart from age that are responsible for venous thromboembolism (VTE) include the presence of comorbid conditions, increased risk of falls, renal insufficiency, potential drug interactions, and dementia [27]. The Fit fOR The Aged (FORTA) list is a drug classification combining positive and negative labeling of drugs frequently prescribed to elderly patients [29]. As per the consensus, apixaban has been rated as FORTA “A” (i.e., A-bsolutely = indispensable drug, clear-cut benefit in terms of efficacy/safety ratio proven in elderly patients for a given indication), while warfarin, dabigatran, and rivaroxaban are given FORTA B label (i.e., B-eneficial = drugs with proven or obvious efficacy in the elderly, but limited extent of effect or safety concerns) [30].

Renal impairment

Patients with chronic kidney disease (CKD) are at two to three times higher risk of developing AF as compared to the general population. The risk of thromboembolism due to AF in CKD patients is independently higher as compared to those without CKD [31-33].

**Figure 4:**
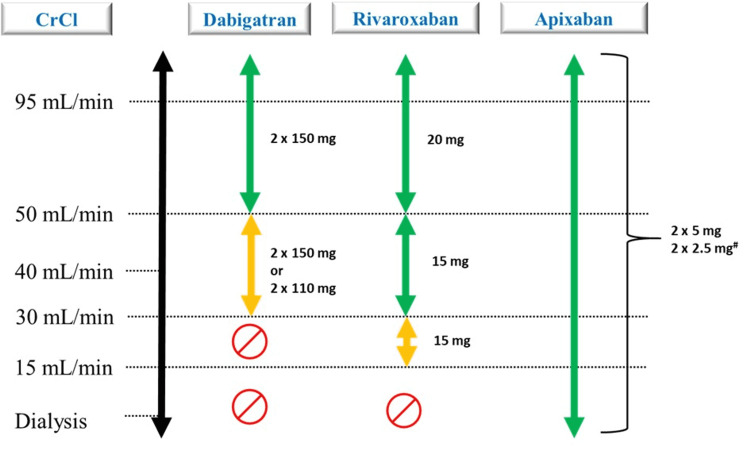
Dose adjustment for CKD patients CrCl: creatinine clearance; EHRA: European Heart Rhythm Association; CKD: chronic kidney disease The figure is adapted from Shroff (2017) [37], Heine et al. (2018) [38], Aursulesei and Costache (2019) [39], Xarelto [40], Eliquis (apixaban) (2012) [36]; Steffel et al. (2021; EHRA Practical Guide) [13].

The NOACs are eliminated renally to varying extent and hence dose adjustment is warranted to manage the risk of bleeding. The calculation of dose reduction is critical as either efficacy or safety is affected with inappropriate doses [34]. Warfarin has been reported to cause renal damage in patients with CKD and is also associated with the progression of renal disease [35]. Apixaban is approved for patients with creatinine clearance <15 mL/min including dialysis for all indications in India [36]. The dosing of NOACs based on stages of CKD has been depicted in Figure 4 [36-40].

Impaired hepatic function

**Figure 5:**
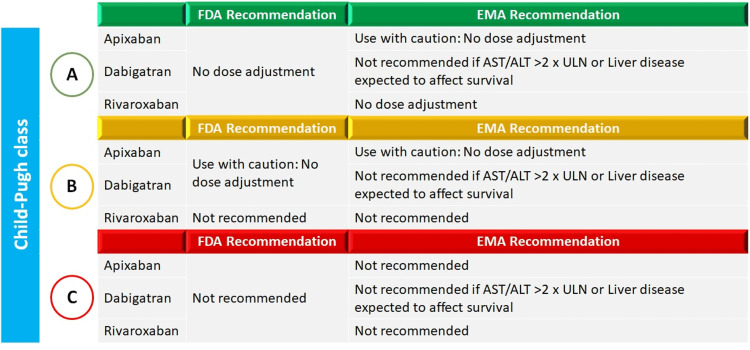
FDA and EMA recommendations for use of NOACs in patients with hepatic impairment ALT: alanine transaminase; AST: aspartate transaminase; EMA: European Medical Agency; FDA: Food and Drug Administration; NOACs: novel oral anticoagulants; ULN: upper limit of normal The figure is adapted from Qamar et al. (2018) [41].

NOACs undergo significant metabolism in the liver. Hepatic impairment thus may lead to an increase in drug levels and decrease in coagulation factors and may result in consequent bleeding. Some NOACs are dependent on cytochrome P450 enzymes for metabolism and in case of hepatic impairment, the activity of these enzymes may be altered. Hence, dose adjustment in patients with hepatic impairment is warranted (Figure 5) [41].

Patients with increased risk of GI bleeding

Dabigatran and rivaroxaban are associated with more than 50% and more than a two-fold increased risk of gastrointestinal (GI) bleeding, respectively, when compared to warfarin [42]. Apixaban has been shown to have comparable major GI bleeds to warfarin. For patients at high risk of GI bleeding, ESC 2016 recommends the use of VKA or NOAC other than dabigatran 150 mg or rivaroxaban 20 mg (class IIa, level B) [20].

Extreme low body weight

The efficacy of NOACs is directly correlated to their plasma concentrations. Since the distribution volume is linked to body weight, extreme body weight can affect their efficacy or safety. Recommendations for dose adjustment based on body weight are represented in Table 5 [43].

**Table 5:** Dose adjustment for NOACs based on body weight NOACs: novel oral anticoagulants The table is adapted from De Caterina and Lip (2017) [43].

NOAC	Lower body weight allowed	Upper body weight allowed	Recommended dose adjustment
Dabigatran etexilate	50 kg	110 kg	No dose adjustment necessary
Rivaroxaban	None	None	No dose adjustment necessary
Apixaban	None	None	No dose adjustments required unless ABC criteria are met.

Minor procedures

**Figure 6:**
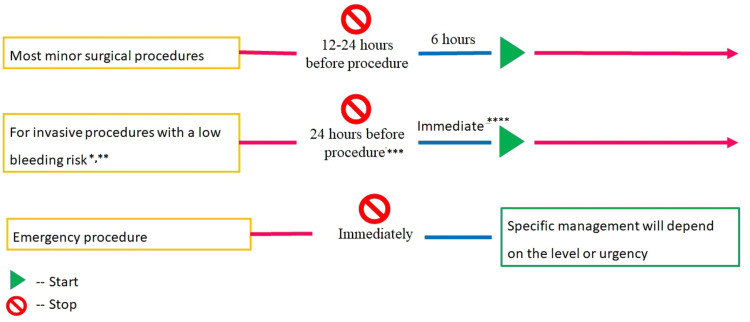
Use of anticoagulants in case of minor procedures NOACs can be resumed six to eight hours after the end of the intervention. *Including cardiac device implantations. **A graded interruption should be considered for patients on dabigatran and a CrCl <80 mL/min. ***Twenty-four hours before in patients with normal kidney function. ****With complete hemostasis. NOACs: novel oral anticoagulants The figure is adapted from Steffel et al. (2021; EHRA Practical Guide) [13].

The EHRA has put together recommendations for temporarily discontinuing anticoagulants for patients undergoing minor invasive procedures (Figure 6) [13,44]. Due to the increased risk of bleeding, anticoagulant therapy may require temporary cessation in some patients. The conditions and timings for cessation and re-initiating anticoagulants depend on the type of procedure and patient characteristics.

The decision of when to stop the NOACs therapy before the invasive procedure is dependent on renal function, type of surgery, and the risk of bleeding. In case a minor surgical intervention which is associated with minimal bleeding risk and/or adequate local hemostasis can be practiced, NOAC therapy can be restarted after six hours. Recommendations regarding restarting NOACs after invasive procedures with low-risk or high-risk bleeding are presented in Table 6 [45].

**Table 6:** Last intake of NOACs before an invasive procedure CrCl: creatinine clearance; NOACs: novel oral anticoagulants The table is adapted from Chiang et al. (2017) [45].

CrCl	Dabigatran		Apixaban, rivaroxaban	
	Low risk	High risk	Low risk	High risk
≥80 mL/min	≥24 h	≥48 h	≥24 h	≥48 h
50−79 mL/min	≥24 h	≥48 h	≥24 h	≥48 h
30−49 mL/min	≥48 h	≥96 h	≥24 h	≥48 h

Caution should be exercised while using NOACs, especially in patients with co-morbidities. The recommendations for the tailor-made for individual patients with NVAF are summarized in Table 7 [27,36,46].

**Table 7:** Selection of NOACs for stroke prevention AF: atrial fibrillation; CKD: chronic kidney disease; GI: gastrointestinal; NOAC: novel oral anticoagulant; NSAID: non-steroidal antiinflammatory drug The table is adapted from Kundu et al. (2016) [27], Eliquis (apixaban) (2012) [36], and Diener et al. (2017) [46].

Recommendation	
Please consider	Prior to beginning NOAC therapy, patient’s cognitive function, level of dependence, mobility, possible issues with drug compliance, and risk of falls must be assessed
	Avoid multiple medications wherever possible
	If a patient is taking non-steroidal antiinflammatory drugs (NSAIDs) switch to another analgesic
	Renal function should be checked before initiating NOAC therapy and thereafter at least once every 8-12 months
	Dose adjustments for specific NOACs should be made depending on the patient’s age, body weight, and renal function
Patients with high risk of gastrointestinal (GI) bleeding	First choice: for patients with a high risk of gastrointestinal bleeding, apixaban 5 mg twice daily or dabigatran 110 mg twice daily may be used; second choice: dabigatran 150 mg twice daily, or rivaroxaban 20 mg once daily
Patients with renal impairment	First choice: patients with AF and stage III CKD (creatinine clearance 30–49 mL/min) may be treated with apixaban 5 mg twice daily (apixaban 2.5 mg twice a day if ≥1 additional criteria: age ≥80 years, body weight ≤60 kg, serum creatinine ≥ 1.5 mg/dL (133 mmol/L are present), rivaroxaban 15 mg daily; second choice: dabigatran 110 mg twice daily; not recommended: dabigatran 150 mg twice daily, rivaroxaban 20 mg once daily
Elderly patients	First choice: in patients older than 75 years, we suggest apixaban 5 mg twice daily (2.5 mg if ≥2 of the following: age ≥80 years, body weight ≤60 kg, or creatinine ≥1.5 mg/dL {133 mmol/L}); second choice: dabigatran 110 mg twice daily, or rivaroxaban 20 mg once daily
Previous history of stroke	First choice: NOACs are preferred over warfarin for secondary stroke prevention in patients with AF

Management of bleeding

The panelists of the advisory board also opined that CPs who are treating AF patients with oral anticoagulants (OACs) in case of a bleeding event should immediately stop the anticoagulant, look for potential drug-drug interactions, and refer the patients to a specialist.

**Figure 7:**
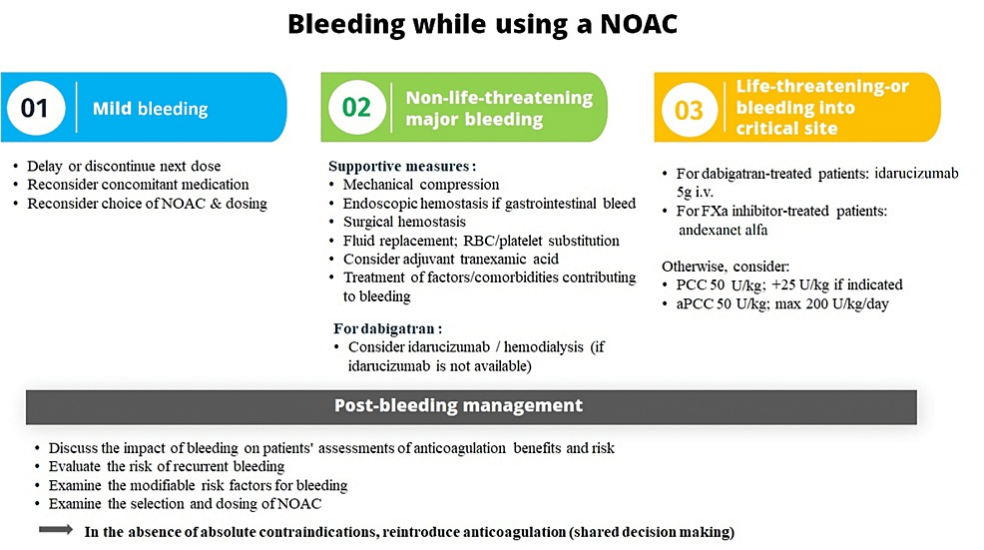
Management of bleeding in patients taking NOAC aPCC: activated prothrombin complex concentrates; NOAC: novel oral anticoagulant; PCC: prothrombin complex concentrates; RBC: red blood cell; WBC: white blood cell The figure is adapted from Steffel et al. (2021; EHRA Practical Guide) [13].

The factor concentrates recommended for the management of bleeding due to NOACs include prothrombin complex concentrate (PCC) and activated PCC (aPCC) [13]. For the reversal of direct NOACs, the DCGI has approved one specific antidote: idarucizumab for dabigatran (Figure 7).

Evaluation of renal function should be considered annually or on a more frequent basis depending on medical history or age as per following EHRA recommendations (Table 8) [13].

**Table 8:** Follow-up of AF patients on NOACs AF: atrial fibrillation; NOAC: novel oral anticoagulant The table is adapted from Steffel et al. (2021; EHRA Practical Guide 2021) [13].

Patient type	Interval
Patients other than those specified below	Yearly
≥ 75 years (especially if on dabigatran) or frail	4-monthly
If renal function CrCl ≤60mL/min: recheck interval = CrCl/10 months	x-monthly
If intercurrent condition may impact renal or hepatic function	If needed

For patients with prior unprovoked bleeding, or experienced warfarin-associated bleeding, or are at a high risk of bleeding, the use of apixaban or dabigatran 110 mg has been recommended since less major bleeding events are associated with them as compared to warfarin [47].

Counseling

**Figure 8:**
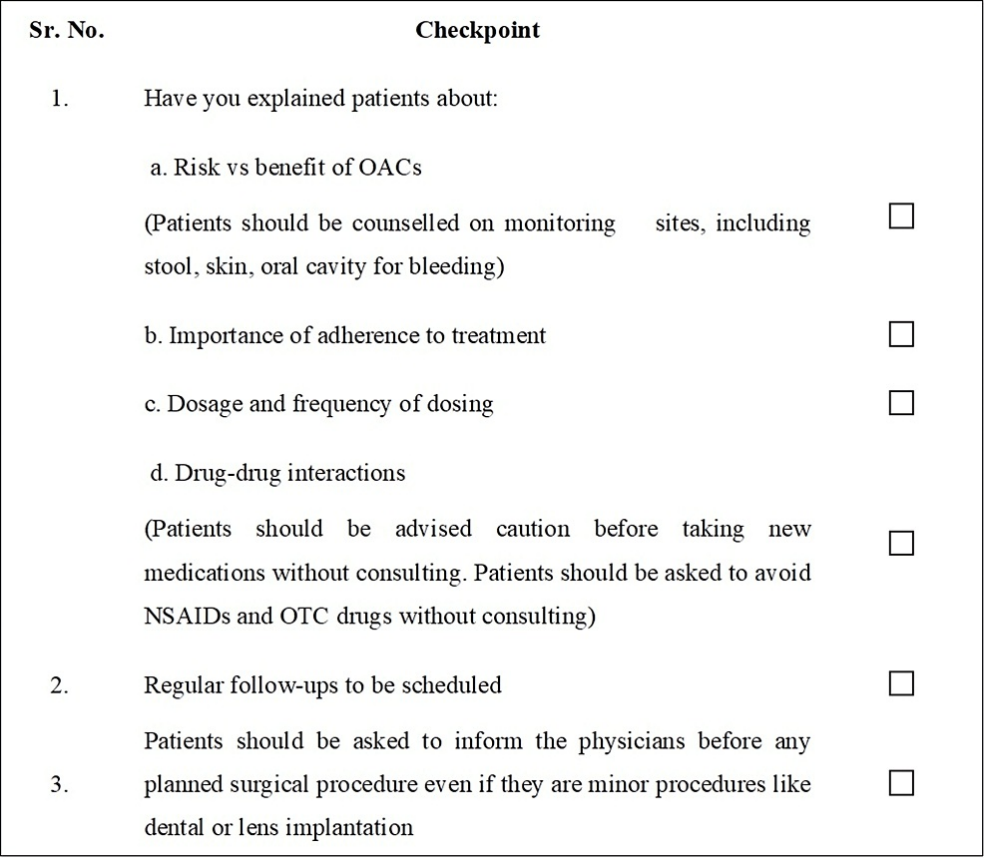
Checklist for patient counseling NSAID: non-steroidal antiinflammatory drug; OAC: oral anticoagulant; OTC: over-the-counter

Patients starting on any OACs should be counseled and properly educated on compliance and precautions related to the therapy [48]. Patient counseling helps in ensuring adherence to the dosing schedule and regular follow-up. The panel on the advisory board discussed the importance of counseling and reached a consensus to develop a checklist that CPs can follow as counseling checkpoints (Figure 8).

Switching

**Figure 9:**
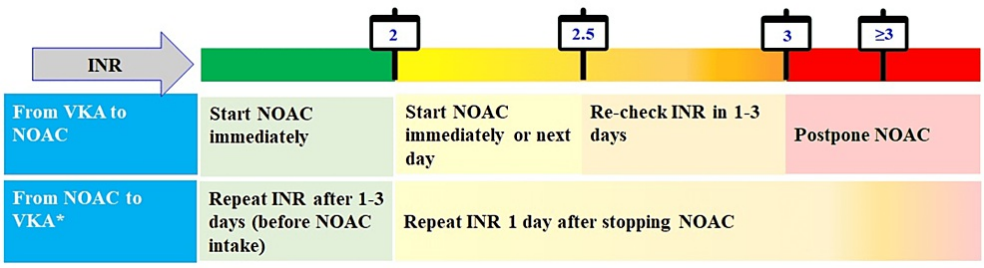
Switching between therapies *Continue NOAC if INR <2 (half dose if on edoxaban), start VKA (loading dose usually used for phenprocoumon). Continue intensive INR sampling for one month, goal: ≥3 consecutive INR values between 2.0 and 3.0. Edoxaban is not approved in India. INR: international normalized ratio; NOAC: novel oral anticoagulant; VKA: vitamin K antagonists The figure is adapted from Steffel et al. (2021; EHRA Practical Guide) [13].

Switching from one oral anticoagulant therapy to another is decided by the treating physician and is dependent on the patient’s eligibility [49]. The major reason for switching from a VKA to a NOAC was occurrence of stroke. Occurrence of myocardial infarction or gastrointestinal bleeding after NOAC initiation significantly increased the chances of switching to VKA. The likelihood of switching from one NOAC to another increased in case of stroke, myocardial infarction, and gastrointestinal bleeding [50]. Ensuring continuous anticoagulant therapy with minimal bleeding risk is paramount while switching between therapies (Figure 9) [13,44].

Algorithm

**Figure 10:**
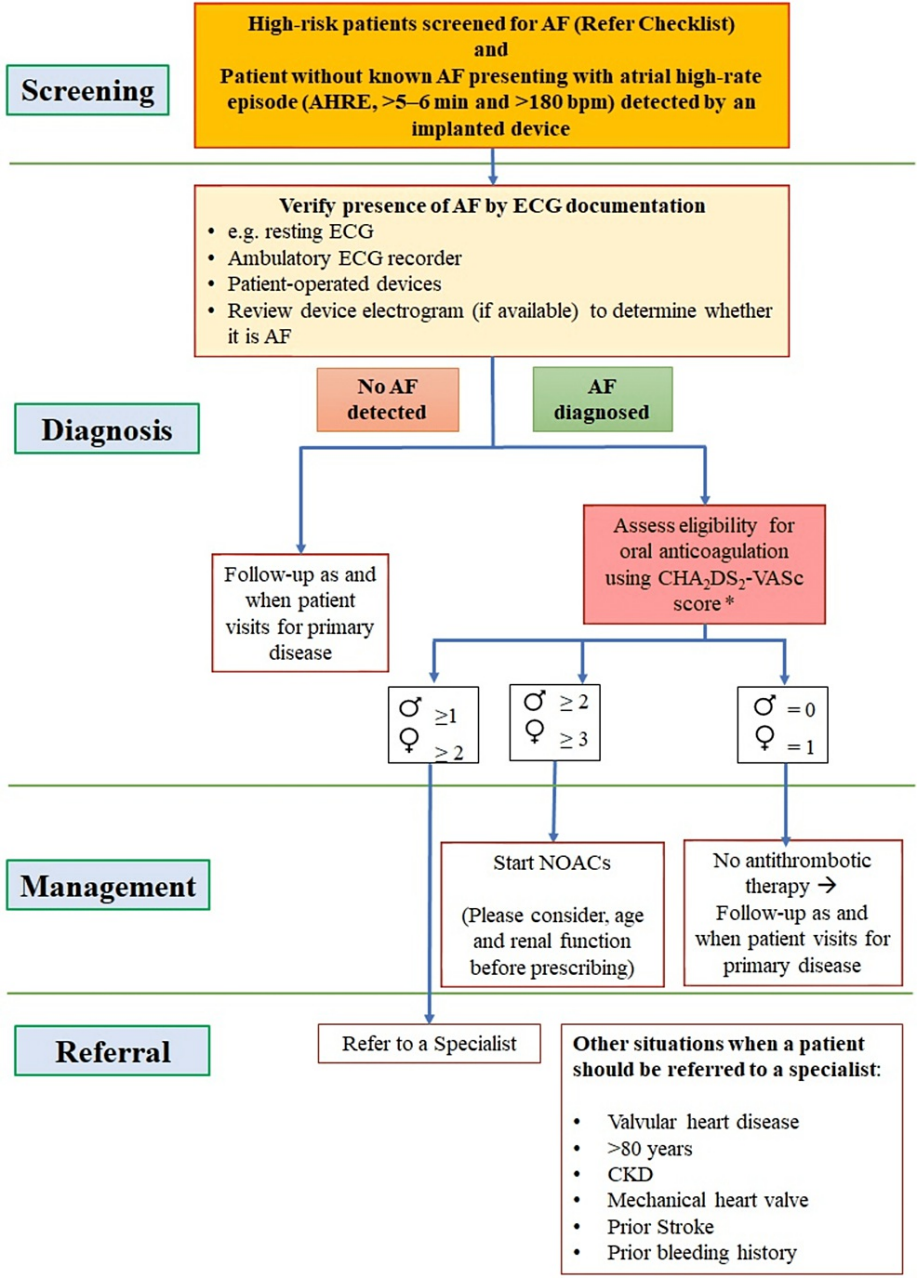
Algorithm for stroke prevention in AF patients *Additionally assess HAS-BLED score AF: atrial fibrillation; AHRE: atrial high-rate episodes; bpm: beats per minute; CHA2DS2-VASc: congestive heart failure, hypertension, age ≥ 75 (doubled), diabetes, stroke (doubled), vascular disease, age 65-74, and sex (female); CKD: chronic kidney disease; ECG: electrocardiogram; HAS-BLED: hypertension, abnormal renal and liver function, stroke, bleeding, labile INR, elderly, drugs or alcohol

The experts who participated in the advisory board meeting suggested the development of a simplified algorithm that CPs can prevent stroke in AF patients. The algorithm provides a comprehensive summary of how to screen, diagnose, manage, and refer patients. The algorithm has been developed based on treatment guidelines, scientific literature, and clinical experience of the experts (Figure 10).

## Conclusions

The CPs being the primary point of contact for patients, need to be equipped for taking quick decisions regarding the need, choice, and dose of oral anticoagulants to reduce the risk of stroke/systemic embolism in patients with NVAF. In the advisory board convened, the experts came up with a simplified protocol based on the current guidelines of stroke prevention completed with simple checklists as ready reckoner for CPs to refer to. The ultimate goal is to empower the CPs to prescribe NOACs to patients with NVAF.
